# Biallelic Variant, c.644-13_644-9del in *UNC50* Is Associated With Congenital Myasthenia Syndrome

**DOI:** 10.1002/ajmg.a.64086

**Published:** 2025-04-12

**Authors:** Mangalore S. Shravya, Greeshma Purushothama, Periyasamy Radhakrishnan, Malavika Hebbar, Shyamala Guruvare, Mary Mathew, Gandham SriLakshmi Bhavani, Shruti Bajaj, Katta M. Girisha, Anju Shukla, Shalini S. Nayak

**Affiliations:** 1 Department of Medical Genetics, Kasturba Medical College, Manipal, Manipal Academy of Higher Education, Manipal, India; 2 Department of Obstetrics and Gynecology, Kasturba Medical College, Manipal, Manipal Academy of Higher Education, Manipal, India; 3 Department of Pathology, Kasturba Medical College, Manipal, Manipal Academy of Higher Education, Manipal, India; 4 The Purple Gene Clinic, Mumbai, India; 5 Department of Genetics, College of Medicine and Health Sciences, Sultan Qaboos University, Muscat, Sultanate of Oman

**Keywords:** arthrogryposis multiplex congenita, congenital myasthenia syndrome, fetal akinesia deformation sequence, neuromuscular disease, perinatal autopsy, UNC50

## Abstract

*UNC50* encodes a transmembrane protein that plays a crucial role in L-acetylcholine receptor trafficking and thus cholinergic transmission at the neuromuscular junction. Previously, a biallelic loss-of-function variant in *UNC50* was reported in an individual with lethal arthrogryposis multiplex congenita. We herein describe affected individuals from two unrelated families with arthrogryposis multiplex congenita in one family and a severe early-onset neuromuscular dysfunction in the other, both within the spectrum of congenital myasthenia syndrome. A biallelic variant, c.644-13_644-9del, p.? in intron 5 of *UNC50* (NM_014044.7) was identified in both families. Transcript analysis in the peripheral blood cDNA of the heterozygous carrier parents of family 1 revealed that the c.644-13_644-9del variant leads to aberrant splicing. With these findings, we validated the association of disease-causing variants in *UNC50* with congenital myasthenia syndrome.

## 1 Introduction

*UNC50*, also known as Unc-50 inner nuclear membrane RNA binding protein, encodes a transmembrane protein that localizes to the Golgi apparatus and is conserved from yeast to humans. The function of Unc50 was largely revealed through genetic screens that were performed in the nematode *Caenorhabditis elegans* for the identification of functional acetylcholine receptors (AChRs) and molecules required for AChR assembly. UNC50 is known to play a crucial role in L-acetylcholine receptor trafficking and thus in cholinergic transmission at the neuromuscular junction (NMJ) (Eimer et al. 2007).

To date, there is a single report of a family with a biallelic variant, c.750_751del; p.(Cys251PhefsTer4), in exon 6 of *UNC50*, presenting with stillbirth at 28 weeks of gestation with contractures across major joints, muscle atrophy of limbs, and pulmonary hypoplasia (Abiusi et al. 2017). We report the clinical and genomic data of two additional families harboring a biallelic variant in *UNC50*, thus validating and expanding the clinical spectrum of *UNC50*-related disorders.

## 2 Subjects and Methods

### 2.1 Participant Recruitment

We ascertained multiple subjects from two unrelated families with consanguinity [family 1: subject 1 (S1), subject 2 (S2), subject 3 (S3); family 2: subject 4 (S4), subject 5 (S5)] affected by a severe neuromuscular disorder. Informed consent was obtained from both families for inclusion in the research studies, publication of photographs and collection of clinical and genomic data. The studies were approved by the Institutional Ethics Committee of Kasturba Medical College and Hospital, Manipal.

### 2.2 Genomic Testing

#### 2.2.1 Exome Sequencing

The genomic DNA was extracted from fetal liver tissue of S2, peripheral whole blood samples from S5, and parents of both families using a DNeasy blood and tissue kit (Qiagen, Germany). Trio-exome sequencing was performed for S2 and parents using a modified TWIST exome capture kit (TWIST Biosciences, South San Francisco, California). The total number of genes covered were 21,157 with average targeted coverage per sample of 90%–100% bases with at least 10/20× of depth. The total number of bases targeted by this capture kit was 36.73 Mb. Singleton exome sequencing for S5 from family 2 was performed using the Agilent SureSelect Clinical Research Exome v6 capture kit. Annotation was carried out by an in-house pipeline against the GRCh38 version of the human genome. Genomic variants were filtered based on in-house and gnomAD allele frequencies and prioritized based on predicted in silico scores and concordance with the phenotype observed in the affected individuals (Kausthubham et al. 2021). Homozygous regions for a minimum size of 1 Mb from the exome sequencing data of the probands were identified using AutoMap (v1.2) (Quinodoz et al. 2021). To visualize and evaluate the variant, we used the integrative genomics viewer (IGV) (Broad Institute, Cambridge, UK) (Robinson et al. 2017). The variant was classified according to the American College of Medical Genetics and Genomics (ACMG) classification guidelines (Richards et al. 2015).

#### 2.2.2 RNA Isolation and Splice Site Analysis

RNA studies could not be performed on the affected individuals, as they were deceased by the time the genomic results were obtained in both families. Hence, total RNA was isolated from peripheral blood samples of parents from family 1 using the Quixtract total RNA extraction kit (GPDX-RNAprep-50, GenePath DX, Pune, India) and was used for splice site analysis. The total RNA content was assessed by absorbance at 260 nm and purity by A_260/280_ ratios and then reverse transcribed using SuperScript IV VILO Master Mix (Invitrogen) according to the manufacturer’s protocol and converted to cDNA. Reverse-transcription PCR (RT–PCR) was then performed using the primers (Figures 2A and S2a).

## 3 Results

### 3.1 Clinical Description

#### 3.1.1 Family 1

**Figure 1:**
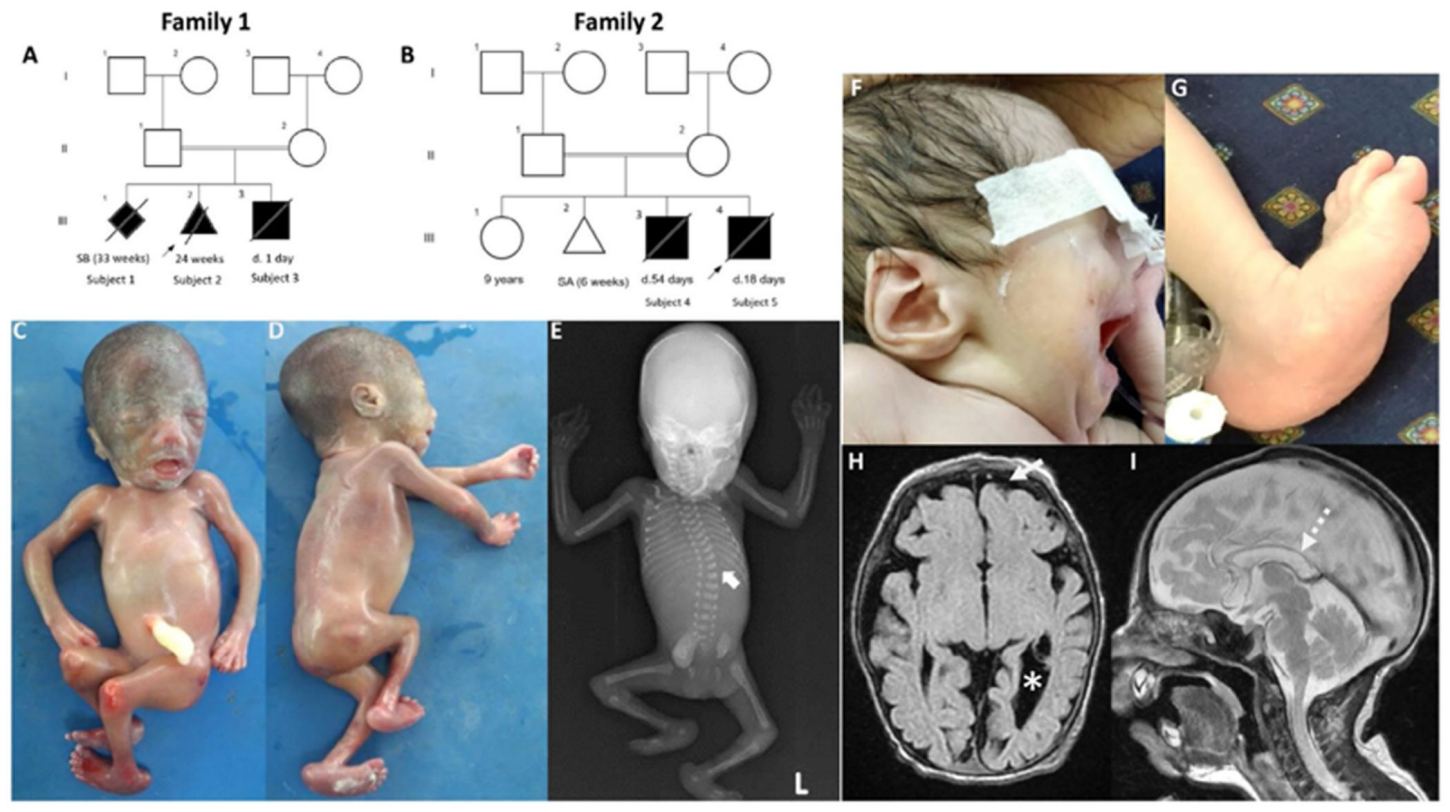
Three generation pedigrees (A and B) and clinical findings (C–I) of families 1 and 2: Subject 2 shows facial dysmorphism, multiple joint contractures, bilateral talipes equinovarus (C and D), and radiograph shows scoliosis (E, white arrow). Subject 5 with facial dysmorphism and rocker bottom feet (F and G). MRI brain depicts cortical volume loss (H-white arrow), prominent ventricles (H-white asterisk) and thin corpus callosum (I-white dotted arrow).

A consanguineously healthy couple with three pregnancies affected by arthrogryposis (Figure 1; Tables 1 and S1). During their first pregnancy (S1), the fetus was noted to have club feet and contractures in the upper and lower limbs on antenatal ultrasonography, which resulted in a stillbirth at 33 weeks of gestation. No further evaluation was performed in S1.

The second pregnancy (S2) was medically terminated at 24 weeks of gestation in view of kyphoscoliosis, constant flexing and crossing of the lower limbs with hyperflexed ankles, and the absence of the right umbilical artery detected via antenatal ultrasonography. Postnatal evaluation of S2 revealed telecanthus, anteverted nares, thin vermilion of the upper lip, contractures across the axillae, hips, knees, and ankles, camptodactyly, and bilateral rocker bottom feet. Radiographs (anterior and lateral views) revealed scoliosis. Histopathological examination of the cerebral cortex, lung, liver, kidney, and muscle was unremarkable. Magnetic resonance imaging of the brain was normal. Multiplex ligation probe-dependent amplification (MLPA) was performed using SALSA MLPA Probemix P060-B2 SMA carrier (MRC, Holland) to rule out type 0 spinal muscular atrophy in S2, which revealed a normal copy number of exons 7 and 8 of the *SMN1* gene.

Antenatal ultrasonography during the third pregnancy (S3) revealed polyhydramnios, reduced fetal movements, intrauterine growth restriction, polyvalvular disease, and severe pleural effusion. This pregnancy resulted in neonatal loss on day one of life. No further information is available for Subject 3.

#### 3.1.2 Family 2

Another consanguineous healthy couple with two consecutive deceased offspring with intrauterine growth retardation, severe neurological dysfunction, rocker bottom feet, severe respiratory insufficiency, and early death was evaluated (Figure 1; Tables 1 and S1). The couple had a healthy 9-year-old female from first conception. The second pregnancy resulted in a spontaneous abortion at 6 weeks of gestation. After an uneventful third pregnancy, a full-term male (S4) with a birth weight of 2.2 kg (−2.18 SD), head circumference of 31 cm (−2.1 SD) and length of 43 cm (−2.7 SD) was born. He had cyanosis and recurrent apnea on day one of life. Microcephaly, a long philtrum, a towering skull (turricephaly) with a closed anterior fontanelle, overlapping fingers, and rocker bottom feet were noted at birth. Metabolic testing and fundus examination were unremarkable. An electroencephalogram (EEG) revealed multifocal epileptiform activity. The ultrasonography of the cranium revealed dilated lateral cerebral ventricles measuring 13 mm. He had to be admitted to the neonatal intensive care unit due to severe apnea and required ventilatory support until day 22 of life. He was discharged on day 28 but was readmitted on day 43 due to recurrent apnea, episodes of posturing, and difficulty in feeding, and succumbed to death on day 54 of life. Karyotype reported a chromosomal pattern of 46,X, inv(Y) (p.11.1q11.23)dpat in S4, inherited from his father. The karyotype of the mother was normal (46,XX).

Subject 5 was born at full term, weighed 1.9 kg (−2.66 SD), had a head circumference of 31.5 cm (−1.93 SD), and a length of 44 cm (−2.3 SD). He presented with a weak cry and was ventilated since day one of life in view of apnea. He had anteverted nares, thin vermilions of the upper and lower lips, a long philtrum, low-set ears, micro-retrognathia, bilateral clinodactyly, and rocker-bottom feet. Deep tendon reflexes were elicitable; however, plantar reflexes were diminished. Ophthalmologic examination, echocardiography, routine hematological and biochemical investigations, and chromosomal microarray did not yield any significant findings. Magnetic resonance imaging of the brain revealed cerebral atrophy, prominent cerebral ventricles, white matter volume loss, and a thin corpus callosum. S5 died on day 18 of life.

### 3.2 Exome Sequencing Identifies a Biallelic Variant, c.644-13_644-9del, in *UNC50* That Leads to Aberrant Splicing

**Figure 2:**
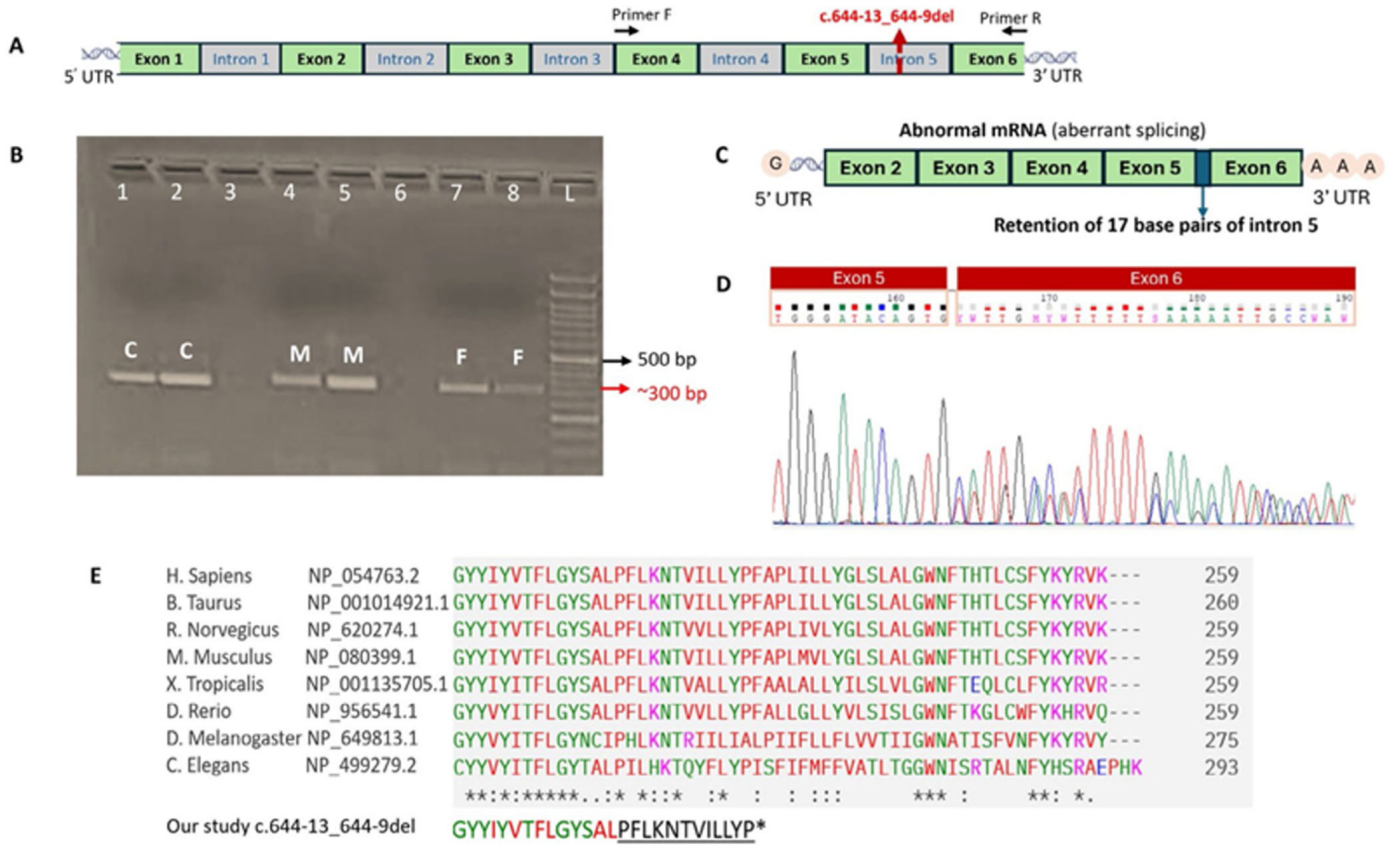
UNC50 mRNA and cDNA synthesis from the blood samples of unaffected parents in family 1 and consequence of variant. (A) *UNC50* contains 6 exons. The variant identified in S2 and S5, c.644-13_644-9del is in intron 5 of *UNC50* (red dotted arrow line). Reverse transcription-polymerase chain reaction (RT-PCR) analysis in blood-derived cDNA from the unaffected parents (F1-II.1 and II.2) and control samples were performed using primers spanning the exon 4 to exon 6 (Primer F and R; black arrow). (B) Gel electrophoresis of the RT-PCR amplicons. The control (C: Lane 1, 2), mother (M: Lane 4, 5), and father (F: 7, 8) samples respectively. All the samples were run in duplicates. The expected product size of RT-PCR amplicons was 290 base pairs, whereas the mother and father samples showed almost the same product size compared to the control. (C) Diagrammatic view of abnormal mRNA of UNC50. The abnormal mRNA shows retention of 17 base pairs of intron 5 in the transcript. (D) Sanger sequencing of RT-PCR amplicons from the parental samples from family 1 showed a retention of 17 base pairs of intron 5 in one of the alleles (heterozygous peaks) and the other allele was wild type. (E) Phylogenetic conservation of the C-terminus of UNC50 homologs via ClustalO alignment. The accession numbers are from the NCBI reference sequence. *Represents a fully conserved residue; : represents residues with strongly similar properties;. represents residues with weakly similar properties. The amino acids that were truncated in the patient are underlined.

Exome sequencing identified a variant, c.644-13_644-9del p.? (NC_000002.12: g.98618155_98618159del) in intron 5 of *UNC50* (NM_014044.7) in a homozygous state in S2 and S5 and in a heterozygous state in the parents of family 1. IGV images of family 1 (S2, mother and father) and family 2 (S5) are provided in Figure S1a. Regions of homozygosity analysis in subject 2 (family 1) and subject 5 (family 2) showed that the variant c.644-13_644-9del (NC_000002.12: g.98618155_98618159del) resides in an autozygous region of 8.13 and 12.92 Mb in subject 2 and subject 5, respectively (Tables S2 and S3). The transcript details of UNC50 are provided in Figure S1b. The chromatograms after Sanger sequencing of the genomic DNA are provided in Figure S1c for family 1. This variant is observed in the gnomAD population database (v4.1.0) in the heterozygous state (variant ID: 2-99234614-ATTCCT-A) with an allele frequency of 0.00007087 and was not detected in our in-house database of 3030 exomes. The in silico splicing tools (Splice AI and Human Splice Finder) predicted the probability of the variant being splice-altering and activation of a cryptic acceptor site. The RT–PCR amplicons obtained from cDNA of the parents of family 1 were nearly identical in product size to those of control (Figure 2A,B). However, Sanger sequencing of these amplicons revealed the retention of 17 bp of intron 5 (c.643_644insTTTGGATTCTTTTCCAG p.Ala215ValfsTer11) in an allele of *UNC50* (Figures 2C, S2b, and S3), and another allele was wild type in the parents of family 1 (Figures 2D,E and S4).

## 4 Discussion

Neuromuscular disorders (NMDs) constitute a group of heterogeneous conditions that affect different components of the motor unit, that is, peripheral nerves, neuromuscular junctions (NMJs) and/or skeletal muscles. The NMJ is a specialized synapse between the motor nerve terminal and the surface of a muscle fiber sarcolemma (Slater 2017). It is a highly complex structure consisting of a presynaptic terminal, a postsynaptic muscle membrane, and a synaptic cleft. The proteins at the NMJs are in these specific compartments and are classified as presynaptic, synaptic, or postsynaptic, and only some of them are ubiquitously expressed (Rodríguez Cruz et al. 2020). Disease-causing variants in genes encoding these proteins disrupt neuromuscular transmission, resulting in muscular weakness of variable severity and onset, and are termed congenital myasthenic syndromes.

Nicotinic acetylcholine receptors (AChRs), which are located in the postsynaptic membrane of NMJs, mediate fast synaptic transmission and modulate neurotransmitter release in the brain (Gotti and Clementi 2004). To be functional, AChRs must be assembled into pentamers and trafficked to the postsynaptic plasma membrane. Malfunction or misexpression of AChRs is involved in several neuropathological processes. Genetic screens performed in the nematode *C. elegans* have contributed to our understanding of the functional AChRs and molecules required for AChR assembly as well as expression (Jones and Sattelle 2004). Unc-50 was identified as one of the significant proteins impacting the surface expression of AChRs (Eimer et al. 2007).

Human *UNC50* contains six exons and encodes a UNC50 protein containing 259 amino acids with a molecular mass of 30 kDa. UNC50 is a Golgi apparatus protein involved in retrograde trafficking of early endosomal vesicles in yeast and in nicotinic receptor trafficking in *C. elegans* (Fitzgerald et al. 2000; Eimer et al. 2007; Fang et al. 2015). It is conserved from yeast to higher eukaryotes (Fang et al. 2015). The protein has two major domains, a topological domain (TP) and a transmembrane domain (TM), in a repetitive pattern. Unc-50 mutants in *C. elegans* display slow and abnormal locomotion called an ‘uncoordinated phenotype’, on which the gene was originally named (Lewis et al. 1980). In these mutants, AChR subunits are expressed normally and are able to assemble and potentially leave the ER but are then misaddressed and transported aberrantly within the cell (Sammi et al. 2017; Selyunin et al. 2017), suggesting that UNC-50 acts at a post assembly step during trafficking of the Lev-AChR to the synapse.

We describe five individuals from two unrelated families with phenotypic findings within the spectrum of congenital myasthenia syndrome. The clinical presentation of arthrogryposis multiplex congenita in family 1 is similar to the phenotype reported previously (Abiusi et al. 2017) of a stillbirth at 28 weeks of gestation, with bilateral club foot, bilateral flexion of the elbows, hips, and knees, bilateral extended wrists, cervical hyperextension, and diffuse subcutaneous edema associated with muscle atrophy of the limb. Additionally, pulmonary hypoplasia and atrophic muscle fibers with areas of myofibrillar disorganization of skeletal muscle were noted on postnatal and histopathological evaluation of this individual (Abiusi et al. 2017). However, in the present study, hematoxylin and eosin staining of S2 did not reveal any significant changes in the muscle. Additionally, in the present study, S4 and S5 presented with severe neuromuscular weakness since birth, manifesting as respiratory insufficiency, mild distal contractures, and early death. Neurological abnormalities such as recurrent opisthotonus posturing appear to be secondary to recurrent apnea and possible cerebral hypoxia. The clinical findings in family 2 expand the phenotypic spectrum of *UNC50-*related disorders beyond arthrogryposis.

The variant identified in the present study, in intron 5 of *UNC50*, causes the activation of a cryptic splice site and leads to the inclusion of 17 base pairs of intron 5 in the altered transcript [c.643_644insTTTGGATTCTTTTCCAG p.(Ala215ValfsTer11)]. This aberration is predicted to shift the amino acid frame from the 215 position and result in premature termination of the protein. The truncated protein is likely to retain 223 of the 259 amino acids of the wild-type protein and thus impair the topological domain (amino acids 209–222) toward the carboxyl end (Abiusi et al. 2017). In a previously reported individual (Abiusi et al. 2017) with a clinical presentation of arthrogryposis multiplex congenita (AMC), a homozygous frameshift deletion in the last exon of *UNC50*, c.750_751del p.(Cys251PhefsTer4), was identified. This variant was predicted to result in truncation of the last nine amino acids toward the carboxyl end of the protein after the last transmembrane domain of *UNC50* (Figures 2E, S4, and S5). These last nine amino acids are highly conserved across the species. Another individual with a heterozygous missense variant of uncertain significance, c.287C > G, p.(Thr96Ser) (NM_014044.5), in *UNC50* has been reported by (Pergande et al. 2020). However, in this cohort of 51 patients from 47 unrelated families aiming to decipher the clinical and genomic landscape of fetal akinesia, no further information on this variant and family has been provided (Pergande et al. 2020). We summarize the clinical and molecular findings observed in the previous and current studies in Tables 1 and S1.

The introduction of the homozygous frameshift deletion identified in *UNC50* by (Abiusi et al. 2017) at an orthologous position in *C. elegans* via CRISPR/Cas9 resulted in an uncoordinated phenotype and a strong decrease in AChR surface expression in the muscle of unc-50 knock-in animals. Additionally, the results of this study indicate that the C-terminal truncation of Unc-50 impairs its function, suggesting that the Unc-50 protein is no longer functional. The study proposed that the *UNC50* variant impairs AChR early during development and emphasized that a refined analysis of *UNC50* and AChR expression at the neuromuscular junction (NMJ) during development could provide critical information about the role of *UNC50* in AChR trafficking at the NMJ in mammals (Abiusi et al. 2017). Unfortunately, due to the early death of the subjects, the lack of availability of further samples, and resource limitations, we were unable to perform any further in vitro or in vivo functional validation of the variant in the present study.

To conclude, this study describes two unrelated families of Indian origin with multiple affected subjects with severe and early-onset neuromuscular disease associated with biallelic variants in *UNC50*. This study broadens the phenotypic and genotypic spectrum of *UNC50*-related disorders and documents *UNC50* as a novel candidate gene for congenital myasthenia syndrome.

## Supplementary Material

Additional supporting information can be found online in the Supporting Information section

Supplementary figures

Supplementary table

## Figures and Tables

**Table 1:** Clinical and molecular profiles of the subjects evaluated in the current and previous studies.

	Abiusi et al. 2017		Present study					
	Family 1		Family 1				Family 2	
	Fetus 1		Subject 1	Subject 2	Subject 3		Subject 4	Subject 5
Age at evaluation	28weeks of gestation		33weeks of gestation	24weeks of gestation	Day 1		54 days	18 days
Outcome	Stillbirth		Stillbirth	Termination of pregnancy	Neonatal loss		Early infantile death	Neonatal death
Consanguinity	Yes		Yes				Yes	
Gender	Male		Male	Female	Male		Male	Male
Major clinical findings	Major joint contractures, pulmonary hypoplasia		Club feet, contractures	Bilateral camptodactyly of fingers, major joint contractures, rocker bottom feet	Antenatally reduced fetal movement		Respiratory insufficiency, overlapping fingers, rocker bottom feet	Respiratory insufficiency, bilateral clinodactyly, decreased plantar reflex, bilateral rocker bottom feet
Imaging of brain	Not done		Not done	Unremarkable	Not done		Dilated lateral ventricles	Corpus callosum thinning, gray and white matter volume loss, prominent lateral ventricles
Overall clinical impression	Arthrogryposis multiplex congenita		Arthrogryposis multiplex congenita				Congenital respiratory insufficiency	
Variant details [NMJ314044.7, *UNC50]*	c.750_751del; p.(Cys251PhefsTer4) Exon 6 (Homozygous)		Not tested	c.644-13_644-9del p.? Intron 5 (homozygous)	Not tested		Not tested	c.644-13_644-9del p.? Intron 5 (homozygous)

## Data Availability

The data that supports the findings of this study are available in the Supporting Information of this article.
