# Supplementary material for: Biallelic Variant, c.644-13_644-9del in UNC50 Is Associated With Congenital Myasthenia Syndrome
Source: Am J Med Genet A. Author manuscript; Available in PMC 2025 Aug 1. (PMC7617616; doi:10.1002/ajmg.a.64086)
Supplement: Supplementary figures [file EMS204848-supplement-Supplementary_figures.docx]

**Supplementary information:**

**Supplementary figures:**

**Supplementary figure 1a:** Integrative Genomics Viewer (IGV) image of family 1 (subject 2, mother and father) and family 2 (subject 5). The IGV image shows that the variant c.644-13_644-9del [NM_014044.7] located in intron 5 of MANE transcript of UNC50 .


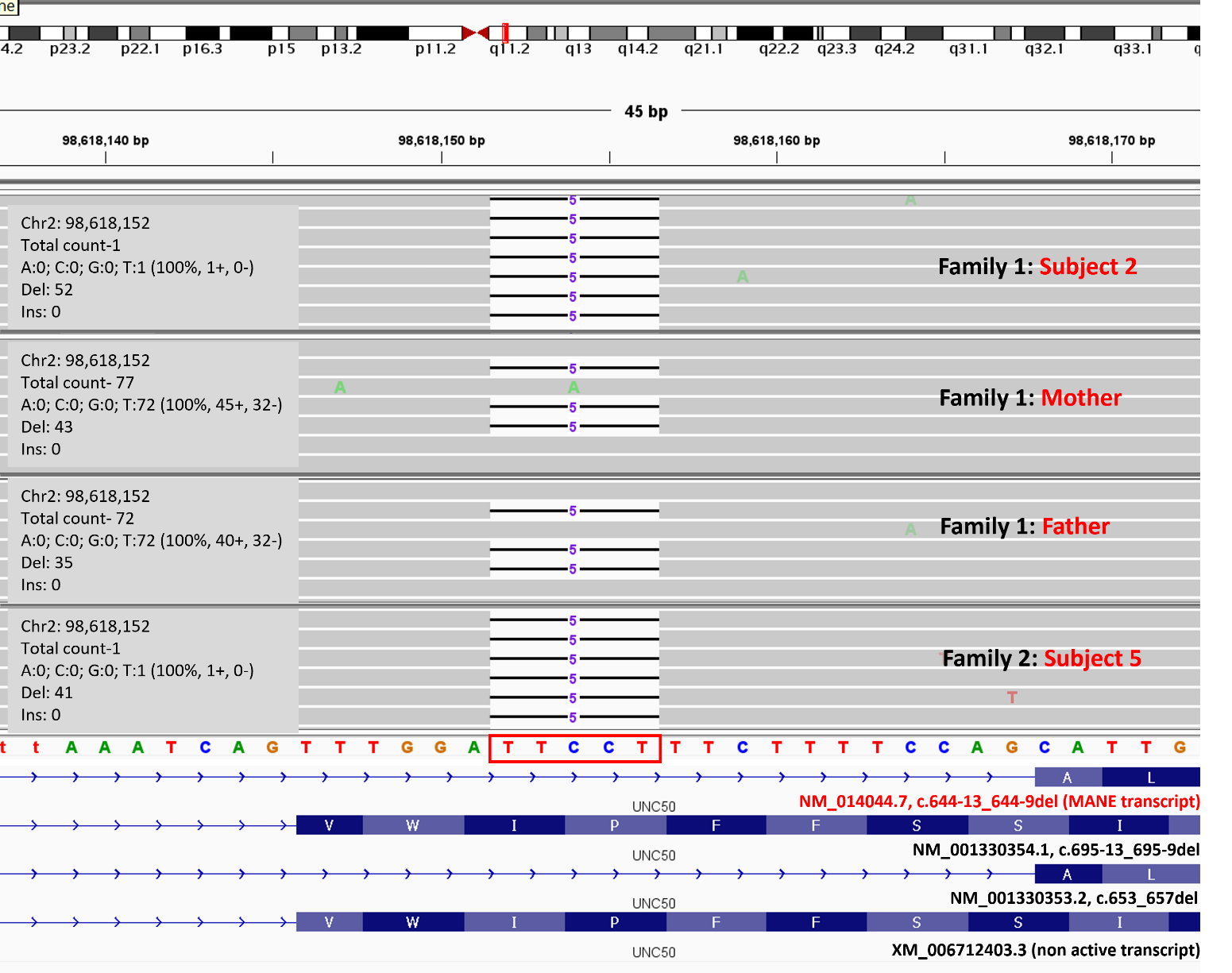


**Supplementary figure 1b:** Depiction of the transcript details of UNC50 (ensemble database)

The black box indicates the active isoforms of UNC50 transcript and NM_014044.7 is the MANE transcript. XM_006712403.4 is an inactive isoform.


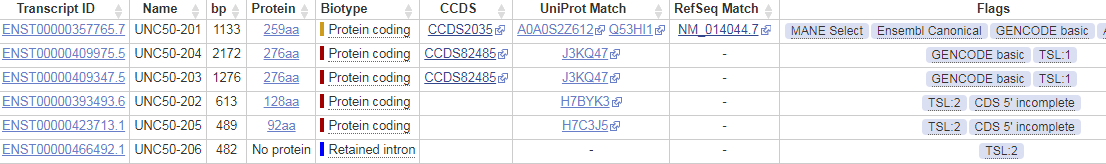

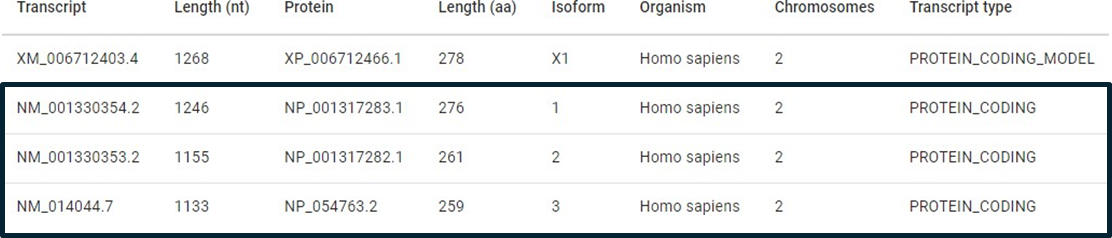


**Supplementary figure 1c:** Chromatograms after Sanger sequencing of the genomic DNA. Sanger sequencing was done in family 1 (subject 2 and parents). The chromatograms were analyzed using chromas software. In family 1, proband showed a homozygous deletion of 5 base pairs of intron 5 of *UNC50* and the parents were heterozygous carriers for the same variant.

Exome sequencing for subject 5 from family 2 was performed at an external laboratory. Unfortunately, during the variant validation process, we were unable to obtain the DNA sample as the subject 5 was deceased. Additionally, segregation analysis for family 2 could not be performed due to the unavailability of parental samples.

**Family 1: Subject 2 -** black arrow indicates the homozygous deletion of 5 bases (CTTTC) of intron 5 of *UNC50* in the proband.


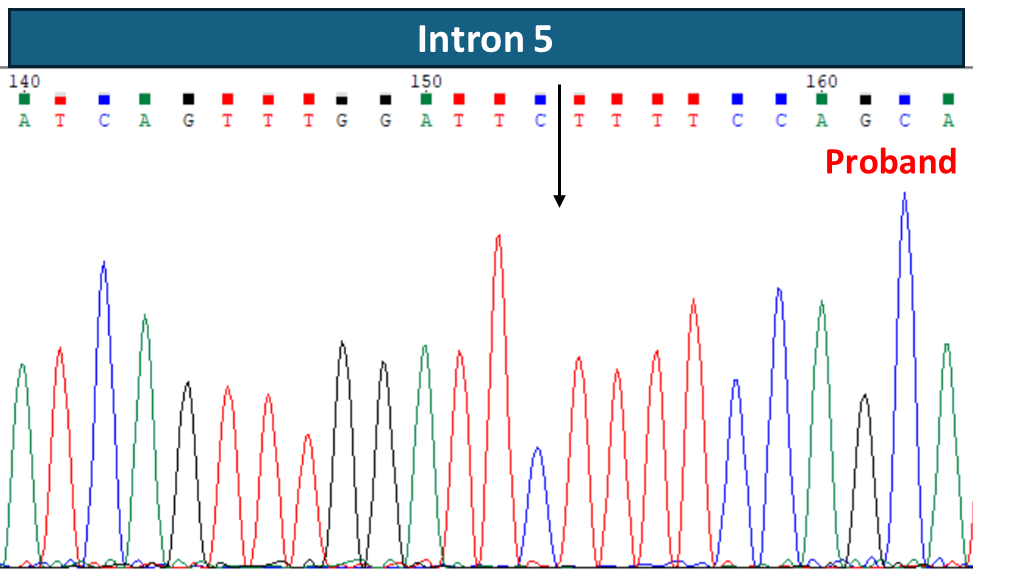


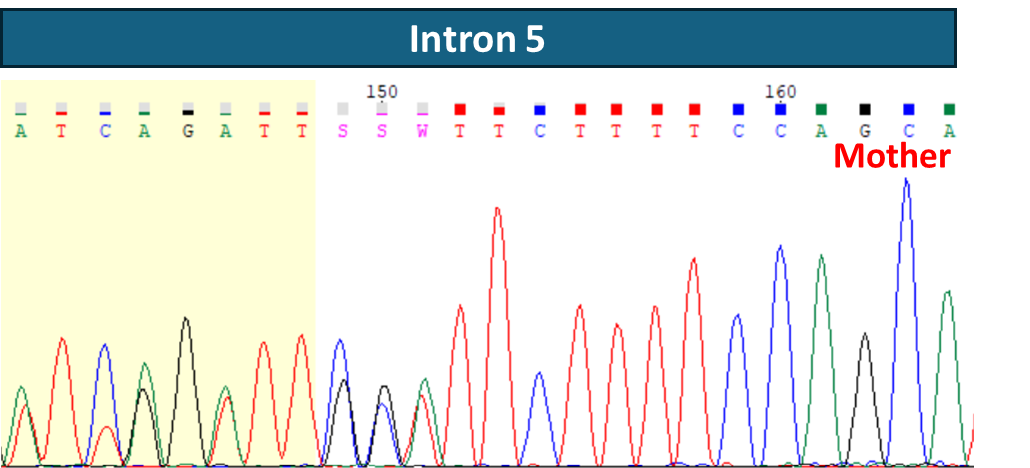


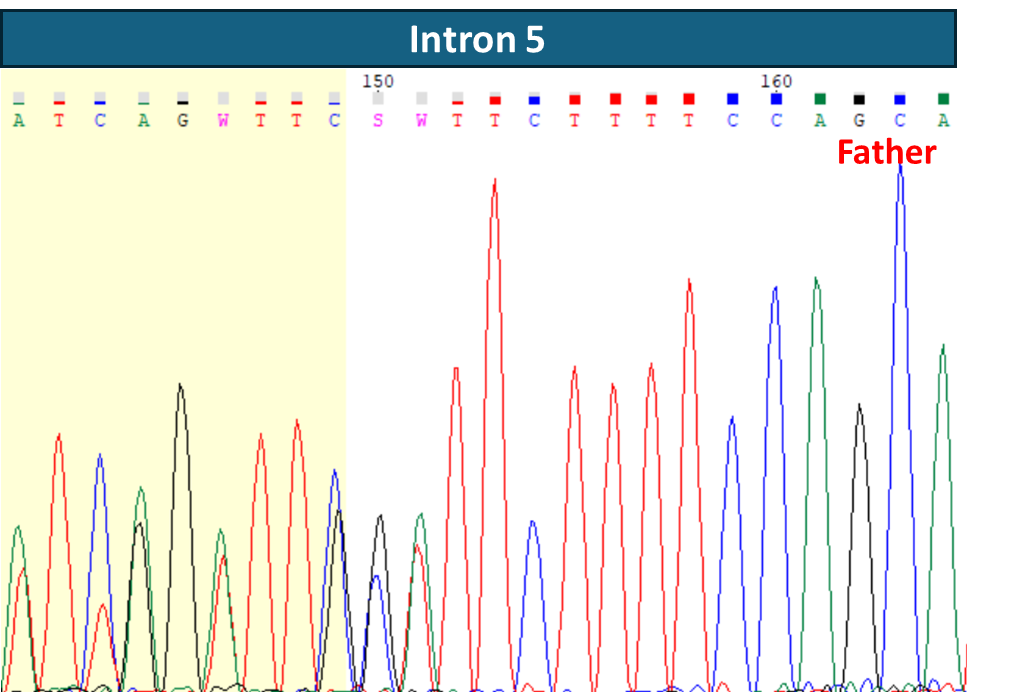


**Supplementary figure 2a:** *UNC50* primer sets

| **Primer set 1** | |
| --- | --- |
| Primers | Sequences |
| Forward primer (Primer F) | TGTGGAATGGGGCTATGCTTTTGA |
| Reverse primer (Primer R) | CACAATACTGTTGACACAGTTACGA |
| **Primer set 2** | |
| Forward primer | TGTGGAATGGGGCTATGCTT |
| Reverse primer | GGCACTGGGATGGAACTTCA |

We used two sets of primers to validate the variant (spanning from exon 4 to exon 6 and exon 4 to 3 prime UTR region). We used agarose gel of 3% and the molecular ladder of 50 base pair. The PCR amplicons obtained from both the primers are of the same size. The primer F and primer R is mentioned in Figure 2A.

**Supplementary figure 2b:** Chromatograms after Sanger sequencing of the RT-PCR amplicons. Sanger sequenced RT-PCR amplicons of control, mother and father from family 1. The chromatograms were analyzed using chromas software. A retention of 17 base pairs of intron 5 in one of the allele (heterozygous peaks) was noted in mother and father and the other allele was wild type in both of them.

**Control:** Wild type (~300 bp amplicon)


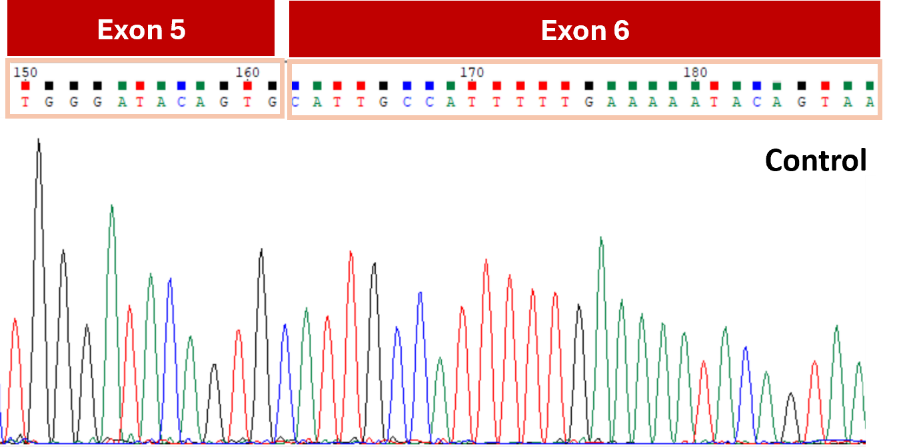


**Parents:** Retention of 17 bp of intron 5 in one of the allele (heterozygous peaks; ~300 bp amplicon)


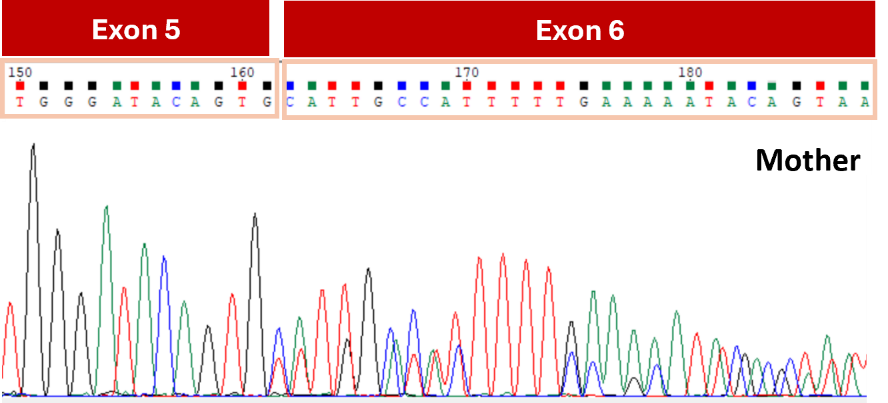


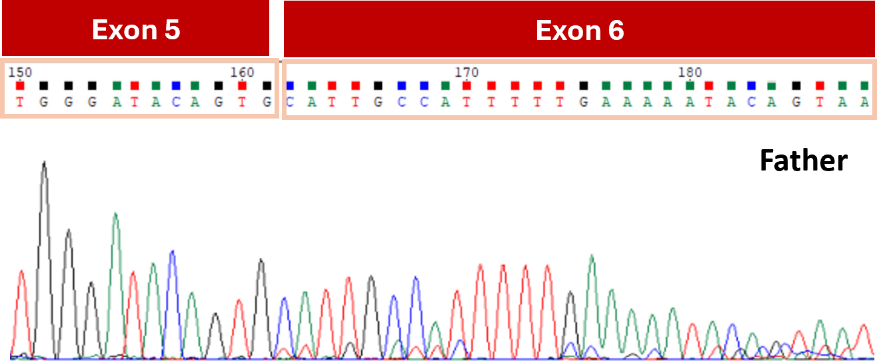


**Ref cDNA Seq: CTGGGATACAGTGCATTGCCATTTTTGAAAAATACAGTAATTCTTCTGTATC**

**cDNA Allele 1: CTGGGATACAGTGCATTGCCATTTTTGAAAAATACAGTAATTCTTCTGTATC**

**cDNA Allele 2: CTGGGATACAGTG[TTTGGATTCTTTTCCAG]CATTGCCATTTTTGAAAAATAC**

Square bracket indicates the insertion of 17 base pairs of intron 5 of *UNC50* to the transcript; exon 4 (**CTGGGATACAGTG);** exon 5 (**CATTGCCATTT….)**

**NM014044.7: c.643_644insTTTGGATTCTTTTCCAG p.(Ala215ValfsTer11)**

**Supplementary figure 3:** The schematic representation of the predicted consequences of the Sanger sequencing of the RT-PCR amplicons from the proband (subject 2 and 5; homozygous peaks) is shown based on the parents’ data.


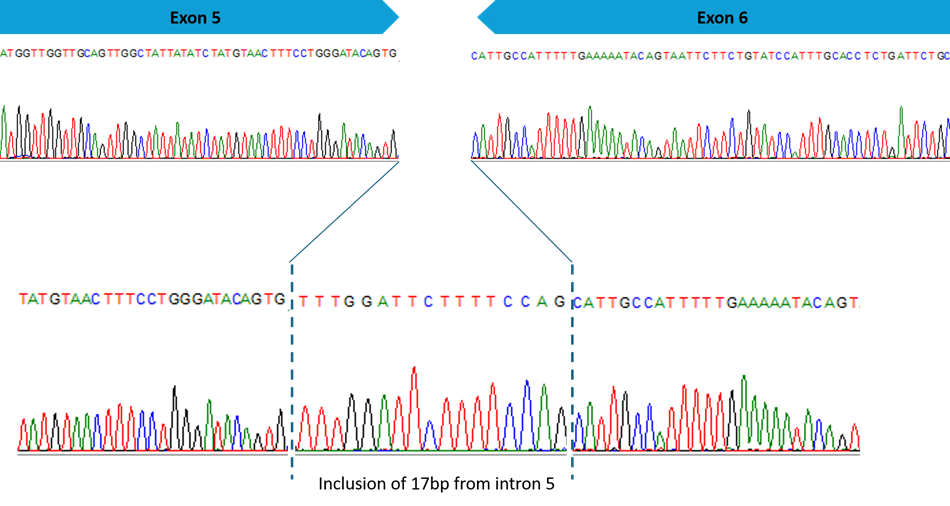


**Supplementary figure 4:** UNC50 amino acid sequence

The image depicts the wild type of amino acid sequence and a mutated amino sequence due to the 17 bp retention of intron 5 (c.643_644insTTTGGATTCTTTTCCAG) in the main sequence likely results in shift in the frame and premature termination of protein sequence p.(Ala215ValfsTer11) affecting highly conserved last nine amino acid sequence of UNC50


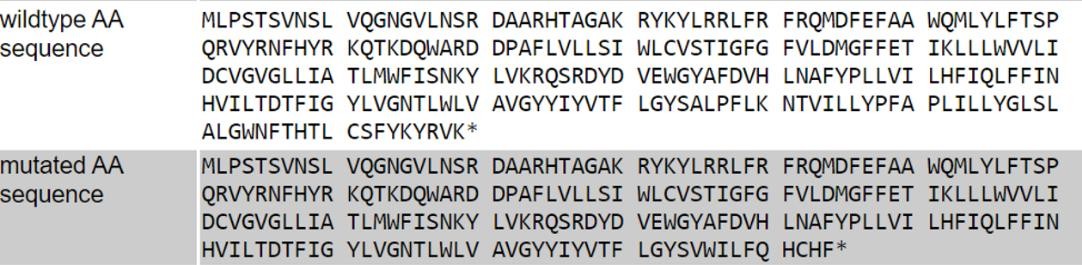


**Supplementary figure 5:** Protein domains of UNC50

UNC50 protein contains 259 amino acids. It has two major domains TP: Topological domain and TM: Transmembrane domain in a repetitive pattern. The variant reported earlier c.750_751del p.Cys251PhefsTer4 and in present study c.643_644insTTTGGATTCTTTTCCAG p.(Ala215ValfsTer11) in the UNC50 are located in the topological domain at the carboxyl end of the protein.

**
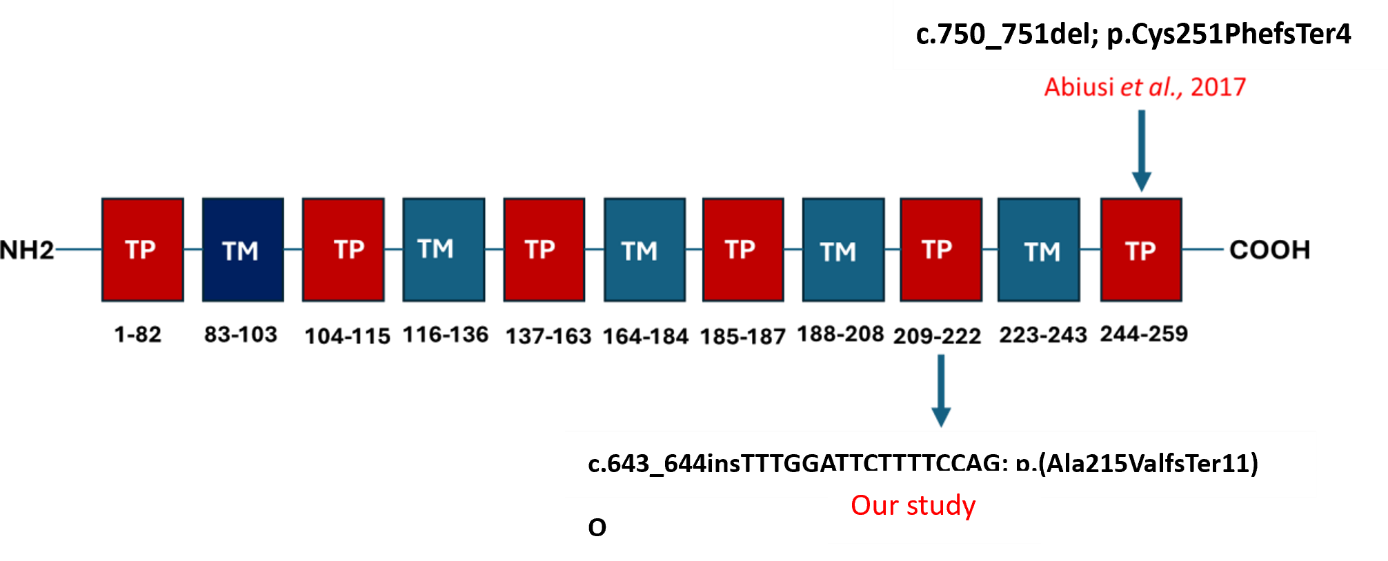
**
