# Supplementary material for: Biallelic Variant, c.644-13_644-9del in UNC50 Is Associated With Congenital Myasthenia Syndrome
Source: Am J Med Genet A. Author manuscript; Available in PMC 2025 Aug 1. (PMC7617616; doi:10.1002/ajmg.a.64086)
Supplement: Supplementary table [file EMS204848-supplement-Supplementary_table.docx]

**Supplementary table 1: Detailed clinical and molecular profile of subjects in the present and previous studies**

|  | Abiusi *et al*., 2017 | Present study | | | | |
| --- | --- | --- | --- | --- | --- | --- |
|  | **Family 1** | **Family 1** | | | **Family 2** | |
|  | **Fetus 1** | **Subject 1** | **Subject 2** | **Subject 3** | **Subject 4** | **Subject 5** |
| Gestation (in weeks) / age (in days) | 28 weeks | 33 weeks | 24 weeks | Day 1 of life | 54 days of life | 18 days of life |
| Outcome | Stillbirth | Stillbirth | Medical termination of pregnancy | Neonatal loss | Infant loss | Neonatal loss |
| Consanguinity | Yes | Yes | | | Yes | |
| Gender | Male | Male | Female | Male | Male | Male |
| Prenatal ultrasound findings | Increased nuchal translucency, cervical hyperextension, and diffuse subcutaneous edema | Club feet and contractures, ?neural tube defect | Abnormal curvature of spine both anteroposteriorly and transversely, constantly flexed and crossed lower limbs with hyper flexed ankles, absent right umbilical artery | Polyhydramnios, reduced fetal movements, intrauterine growth restriction, congenital polyvalvular disease, severe pleural effusion, postpartum hemorrhage | Not available | Not available |
| Clinical features | Bilateral flexion of elbows,  extended wrists,  flexion of hips, knees, club feet,  muscle atrophy of limb, pulmonary hypoplasia | Not available | Telecanthus, anteverted nares and thin vermilion of upper lip, camptodactyly of fingers, contractures across axillae, hips, knees, ankles, bilateral rocker bottom feet | Not available | Microcephaly, long philtrum, towering of skull (turricephaly) with a possibly closed anterior fontanelle, overlapping fingers, rocker bottom feet | Anteverted nares, thin vermilion of upper and lower lips, long and smooth philtrum, left ear longer than right, low set ears, micro-retrognathia, bilateral clinodactyly, decreased plantar reflex,  bilateral rocker bottom feet |
| Neonatal presentation | Not applicable | Not applicable | Not applicable | Not available | Recurrent apnea and cyanosis, ventilated since day 1 of life in view of apnea, arching of the body, paroxysmal movements, and regurgitation of feed | Weak cry, ventilated since day 1 of life in view of apnea |
| Fetogram | Not done | Not done | Scoliosis | Not done | Not done | Not done |
| Imaging of brain | Not done | Not done | Unremarkable | Not done | Dilated lateral ventricles | Corpus callosum thinning, diffuse underdeveloped white matter, and prominent occipital and temporal horns of the lateral ventricles |
| Histopathology | Atrophic muscle fibers and areas of  myofibrillar disorganization | Not done | Unremarkable | Not done | Not done | Not done |
| Disease/condition | Arthrogryposis multiplex congenita | Arthrogryposis multiplex congenita/neuromuscular disease | | | Congenital respiratory insufficiency | |
| Variant details [NM_014044.7, *UNC50*] | c.750_751del; p.Cys251PhefsTer4 in exon 6 (homozygous);  frameshift deletion | No genetic study | c.644-13_644-9del p.? in intron 5 (homozygous) | No genetic study | No genetic study | c.644-13_644-9del p.? in intron 5 (homozygous) |

**Supplementary table 2:** Regions of homozygosity in the subject 2 (family 1) and subject 5 (family 2). The variant c.644-13_644-9del (NC_000002.12: g.98618155_98618159del) resides in an autozygous region (2:98250312-106376496) of 8.13 Mb in subject 2: family 1 and in autozygous region (2:97167729-110091645) of 12.92 Mb in subject 5: family 2.

| **Subject 2 (Family 1)** | **#Chr** | **Begin** | **End** | **Size (Mb)** | **Nb_variants** | | **Percentage_homozygosity** | **Subject 5 (family 2)** | **#Chr** | **Begin** | **End** | **Size (Mb)** | **Nb_variants** | **Percentage_**  **homozygosity** |
| --- | --- | --- | --- | --- | --- | --- | --- | --- | --- | --- | --- | --- | --- | --- |
|  | Chr1 | 23520972 | 26031165 | 2.51 | | 30 | 100.00 |  | Chr1 | 157544307 | 165563768 | 8.02 | 90 | 96.67 |
|  | Chr1 | 63534197 | 107617638 | 44.08 | | 146 | 97.95 |  | Chr1 | 201386633 | 204618112 | 3.23 | 49 | 97.96 |
|  | Chr2 | 11638696 | 36849451 | 25.21 | | 142 | 97.89 |  | **Chr2** | **97167729** | **110091645** | **12.92** | **76** | **94.74** |
|  | **Chr2** | **98250312** | **106376496** | **8.13** | | **35** | **100.00** |  | Chr2 | 141015828 | 169546520 | 28.53 | 102 | 98.04 |
|  | Chr2 | 214809500 | 222922841 | 8.11 | | 80 | 92.50 |  | Chr2 | 233561018 | 236167453 | 2.61 | 37 | 91.89 |
|  | Chr3 | 150703873 | 185519176 | 34.82 | | 122 | 95.90 |  | Chr7 | 101551143 | 121873507 | 20.32 | 54 | 90.74 |
|  | Chr4 | 218771 | 7735410 | 7.52 | | 129 | 97.67 |  | Chr9 | 133778872 | 136954236 | 3.18 | 53 | 88.68 |
|  | Chr5 | 18721381 | 68184176 | 49.46 | | 114 | 95.61 |  | Chr10 | 119819528 | 127012378 | 7.19 | 45 | 93.33 |
|  | Chr5 | 139481561 | 141095172 | 1.61 | | 46 | 89.13 |  | Chr10 | 132348129 | 133568423 | 1.22 | 51 | 88.24 |
|  | Chr5 | 147648367 | 149028538 | 1.38 | | 29 | 89.66 |  | Chr14 | 95204467 | 104730028 | 9.53 | 68 | 97.06 |
|  | Chr5 | 176529515 | 181234980 | 4.71 | | 97 | 95.88 |  | Chr18 | 57668180 | 58981524 | 1.31 | 31 | 93.55 |
|  | Chr6 | 33673602 | 42964462 | 9.29 | | 86 | 95.35 |  | Chr20 | 6116048 | 19830061 | 13.71 | 45 | 97.78 |
|  | Chr6 | 46839072 | 48008847 | 1.17 | | 26 | 92.31 |  |  |  |  |  |  |  |
|  | Chr7 | 136407999 | 141664912 | 5.26 | | 30 | 90.00 |  |  |  |  |  |  |  |
|  | Chr8 | 24489230 | 38057097 | 13.57 | | 57 | 96.49 |  |  |  |  |  |  |  |
|  | Chr8 | 38057207 | 75350301 | 37.29 | | 68 | 94.12 |  |  |  |  |  |  |  |
|  | Chr8 | 123774131 | 144889982 | 21.12 | | 193 | 97.93 |  |  |  |  |  |  |  |
|  | Chr9 | 27205023 | 39078723 | 11.87 | | 97 | 97.94 |  |  |  |  |  |  |  |
|  | Chr9 | 74802056 | 85002350 | 10.20 | | 38 | 92.11 |  |  |  |  |  |  |  |
|  | Chr10 | 19595335 | 46337370 | 26.74 | | 100 | 95.00 |  |  |  |  |  |  |  |
|  | Chr10 | 46550723 | 77634571 | 31.08 | | 145 | 96.55 |  |  |  |  |  |  |  |
|  | Chr11 | 1018175 | 6319295 | 5.30 | | 246 | 97.97 |  |  |  |  |  |  |  |
|  | Chr11 | 92801846 | 106101463 | 13.30 | | 65 | 92.31 |  |  |  |  |  |  |  |
|  | Chr12 | 109445569 | 133234117 | 23.79 | | 234 | 98.72 |  |  |  |  |  |  |  |
|  | Chr13 | 43023729 | 79342838 | 36.32 | | 87 | 93.10 |  |  |  |  |  |  |  |
|  | Chr15 | 32484831 | 41855441 | 9.37 | | 84 | 88.10 |  |  |  |  |  |  |  |
|  | Chr15 | 95784163 | 101051718 | 5.27 | | 28 | 100.00 |  |  |  |  |  |  |  |
|  | Chr16 | 1256926 | 4195692 | 2.94 | | 94 | 94.68 |  |  |  |  |  |  |  |
|  | Chr17 | 41755893 | 44176913 | 2.42 | | 69 | 88.41 |  |  |  |  |  |  |  |
|  | Chr20 | 1611703 | 5942557 | 4.33 | | 75 | 100.00 |  |  |  |  |  |  |  |
|  | Chr22 | 16591593 | 23998535 | 7.41 | | 147 | 96.60 |  |  |  |  |  |  |  |
|  | Chr22 | 24183081 | 28800506 | 4.62 | | 44 | 97.73 |  |  |  |  |  |  |  |

Variant (NC_000002.12: g.98618155_98618159del ) in *UNC50* lies in the highlighted (red) region. chr: chromosome

Family 1 (subject 2): Total regions of homozygosity are 470.2 Mb (autosomal chromosomes)

Family 2 (subject 5): Total regions of homozygosity are 111.77 Mb (autosomal chromosomes)

**Supplementary table 3:** Homozygous block (grey) around the *UNC50* locus, using the exome sequencing data from subject 2 (family 1) and subject 5 (family 2). Haplotype analysis between both the affected probands from family 1 and 2, with the *UNC50* variant c.644-13_644-9del, NC_000002.12: g.98618155_98618159del (red bold).

| **Subject 2 (Family 1)** | **#Chr** | **Genomic position** | **Ref** | **Altered** | **Zygosity** | **Index frequency** | **Index read number** | **Subject 5 (family 2)** | **#Chr** | **Genomic position** | **Ref** | **Altered** | **Zygosity** | **Index frequency** | **Index read number** |
| --- | --- | --- | --- | --- | --- | --- | --- | --- | --- | --- | --- | --- | --- | --- | --- |
|  | chr2 | 85663812 | G | A | Het | 17,19 | 36 |  | chr2 | 79158697 | T | G | Het | 177,46 | 223 |
|  | chr2 | 85698763 | C | T | Het | 75,49 | 124 |  | chr2 | 84699594 | G | T | Het | 21,20 | 41 |
|  | chr2 | 87132066 | G | A | Het | 9,11 | 20 |  | chr2 | 87128723 | T | C | Het | 17,3 | 20 |
|  | Chr2 | 87714200 | G | A | Het | 73,13 | 86 |  | Chr2 | 87128731 | A | G | Het | 17,3 | 20 |
|  | Chr2 | 87785111 | A | G | Het | 20,6 | 26 |  | Chr2 | 88173279 | G | A | Hom | 0,47 | 47 |
|  | Chr2 | 89176509 | C | G | Het | 33,44 | 77 |  | Chr2 | 88452423 | C | A | Hom | 0,7 | 7 |
|  | Chr2 | 90360885 | A | C | Het | 9,2 | 11 |  | Chr2 | 89040316 | T | C | Het | 144, 139 | 283 |
|  | Chr2 | 90360902- 90360923 | CGAGGCGGCGGAGGCATAAAGC | - | Het | 11,2 | 13 |  | Chr2 | 95935488 | G | A | Het | 35, 7 | 42 |
|  | Chr2 | 95594068 | C | T | Het | 46,6 | 52 |  | Chr2 | 96284585 | C | T | Hom | 0, 243 | 243 |
|  | Chr2 | 97556967 | T | C | Het | 32,33 | 65 |  | Chr2 | 97585395 | A | G | Het | 51,9 | 60 |
|  | **Chr2** | **98618152- 98618156** | **TTCCT** | **-** | **Hom** | **0,46** | **46** |  | **Chr2** | **98618152- 98618156** | **TTCCT** | **-** | **Hom** | **0,29** | **29** |
|  | Chr2 | 108247088 | A | G | Het | 20,19 | 39 |  | Chr2 | 107872221 | G | A | Hom | 0, 210 | 210 |
|  | Chr2 | 110672760 | T | C | Het | 37,44 | 81 |  | Chr2 | 108499560 | A | G | Het | 63,21 | 84 |
|  | Chr2 | 112521308 | A | C | Het | 15, 13 | 28 |  | Chr2 | 112662996 | A | G | Hom | 0,70 | 70 |
|  | Chr2 | 119647055 | T | - | Het | 4,6,25 | 35 |  | Chr2 | 113029003 | A | G | Hom | 36, 31 | 67 |
|  | Chr2 | 119946333 | T | C | Het | 22,39 | 61 |  | Chr2 | 121281207 | G | A | Het | 144,155 | 299 |
|  | Chr2 | 120986307 | G | A | Het | 20,20 | 40 |  | Chr2 | 127701592- 127701609 | GCCGCGGAGCCGCTGCTC | - | Het | 16, 26 | 42 |
|  | Chr2 | 127106914 | C | T | Het | 20,28 | 48 |  | Chr2 | 128145957 | C | T | Het | 65, 47 | 112 |
|  | Chr2 | 127885412 | - | GGGGCGGC | Hom | 0,2 | 2 |  | Chr2 | 130964246 | G | A | Het | 2,4 | 6 |
|  | Chr2 | 130115218 | A | G | Het | 89,18 | 107 |  | Chr2 | 131492494 | G | A | Het | 1,4 | 5 |
|  | Chr2 | 130115298 | C | T | Het | 120,22 | 142 |  | chr2 | 144398984 | G | A | Het | 66,97 | 163 |
|  | chr2 | 130155216 | A | C | Het | 56,5 | 61 |  | chr2 | 162144091 | T | C | Hom | 0,72 | 72 |

chr: chromosome, Het: heterozygous, Hom: homozygous
